# Evaluation of coexistence of Alzheimer’s disease in idiopathic normal pressure hydrocephalus using ELISA analyses for CSF biomarkers

**DOI:** 10.1186/1471-2377-14-66

**Published:** 2014-04-01

**Authors:** Tae Sung Lim, Jun Young Choi, Sun Ah Park, Young Chul Youn, Hyun Young Lee, Byung Gon Kim, In Soo Joo, Kyoon Huh, So Young Moon

**Affiliations:** 1 Department of Neurology, School of Medicine, AjouUniversity, 5 San, Woncheon-dong, Yongtong-gu, Suwon-si, Kyunggi-do 442-749, Republic of Korea; 2 Department of Neurology, Soonchunhyang University College of Medicine, Bucheon, Republic of Korea; 3 Department of Neurology, Chung-Ang University College of Medicine, Seoul, Republic of Korea; 4 Regional Clinical Trial Center, Ajou University Medical Center, Suwon, Republic of Korea

**Keywords:** Normal pressure hydrocephalus, Alzheimer’s disease, Cerebrospinal fluid, Lumbar puncture, Neuropsychological tests

## Abstract

**Background:**

We investigated levels of the β-amyloid 1–42 (Aβ42), total tau protein (T-tau) and tau phosphorylated at position threonine 181 (P-tau) in cerebrospinal fluid (CSF) of idiopathic normal pressure hydrocephalus (iNPH) patients and tried to find their clinical implications in the evaluation and treatment of iNPH.

**Method:**

Twenty-five possible iNPH patients were prospectively enrolled and their CSF was collected to analyze levels of Aβ42, T-tau and P-tau using ELISA method. Gait disturbance, urinary incontinence, and cognitive impairment were semi-quantified and detailed neuropsychological (NP) test was performed.

**Result:**

Eight iNPH patients were classified into the lower CSF Aβ42 group and 17 patients were classified into the higher CSF Aβ42 group. There was no difference in the iNPH grading score and its improvement after LP between the two groups. The lower CSF Aβ42 group showed more deficits in attention, visuospatial function and verbal memory in the baseline NP test and less improvement in phonemic categorical naming and frontal inhibitory function after LP.

**Conclusions:**

Our study suggested that concomitant AD in iNPH patients might contribute to lumbar puncture or shunt unresponsiveness, especially in the field of cognitive dysfunction.

## Background

Idiopathic normal pressure hydrocephalus (iNPH) is characterized by a clinical triad of symptoms including cognitive impairment, gait difficulty, and urinary incontinence along with ventricular enlargement in brain imaging [1,2]. It is a potentially reversible cause of cognitive and motor impairment in older adults using ventriculo-peritoneal (VP) shunt or ventriculo-atrial (VA) shunt operation. While treatment with VP shunts is widely used and effective, the complication rate is high [3] and the risk factors for shunt unresponsiveness are poorly understood. A possible contributor to shunt unresponsiveness is the presence of comorbid neurologic conditions that are common in the aged, such as Alzheimer’s disease (AD) [4].

AD and iNPH have different pathomechanism, but recent studies pointed out common pathomechanism between the two diseases [5,6]. However there is still controversy about the clinical implications of the coexistence of AD pathology in iNPH patients. Some suggested that AD was only a bystander and the rate of coexistence of AD was similar to that of normal population [7-9] while the others reported the poor shunt response was possibly due to AD pathology [4,10]. Since the introduction of ELISA method to detect cerebrospinal fluid (CSF) biomarkers for AD, there have been many studies about the differential role of levels of the β-amyloid 1–42 (Aβ42), T-tau protein (T-tau) and tau phosphorylated at position threonine 181 (P-tau) in CSF of iNPH patients [11-15].However, there are still debates about the level of each biomarker in two diseases and the implication of their changes [16,17].

The aim of this study was to investigate levels of the Aβ42, T-tau and P-tau in CSF of iNPH patients and describe the clinical implications in the evaluation and treatment of iNPH.

## Methods

### Participants

Among patients who visited the Department of Neurology at the Ajou Medical Center, Suwon, Korea from March 2010 to February 2012, we consecutively recruited 25 patients who satisfied the criteria for possible iNPH. All patients had had brain MRIs and an LP. The clinical criteria for possible iNPH included following: (1) MRI showing ventricular enlargement (2) Any one symptom from clinical triad (gait disturbance, cognitive deficit or urinary disturbance) which was considered as a symptom compatible with iNPH by the clinician [2]. After making a diagnosis of iNPH, one or two LPs were performed to drain 30 to 50 ml of the CSF and 10 ml of CSF was collected to evaluate biomarkers for AD. The diagnosis of iNPH was made independently from the patients’ response to the LPs.

The CSF of 17 AD patients and 10 normal control subjects which were collected and stored previously at two other hospitals (S.C.H.U.H. and C.A.U.H.) was tested to get a cutoff value of the coexistence of AD. All AD patients met the criteria for probable AD as proposed by the National Institute of Neurological and Communicative Diseases and Stroke and Alzheimer’s disease and Related Disorders Association (NINCDS-ADRDA) [18]. All normal control subjects scored in each cognitive domain test higher than the cutoff value. The cutoff values for each test score were represented as a –1.0 SD below the published norms for their age and education group [19].

We excluded patients with a history of significant hearing or visual impairment that could render interview participation difficult, as well as those with a history of other neurological disorders (e.g., idiopathic Parkinson’s disease, dementia with Lewy bodies, or active epilepsy), psychiatric illnesses (e.g., schizophrenia, mental retardation, major depression, or mania), those taking psychotropic medications, and those with a history of significant alcohol and/or other substance abuse. Each patient provided written informed consent. If patients had impaired decisional capacity, caregivers provided consent and patients provided assent. This study was approved by the Ajou Institutional Review Board.

### Evaluation of iNPH symptoms

An iNPH scale modified from Larsson et al. [20] and Krauss et al. [21] that assessed gait [1 = normal, 2 = walk without any assistive device but insecure, 3 = walk with cane, 4 = walk with bimanual support (walker), 5 = walk aided by an assistant, 6 = wheelchair-bounded], urinary disturbance (0 = normal, 1 = sporadic (1–3 or more times per week but less than once per day) incontinence or urge phenomena, 2 = frequent (1 or more times per day) incontinence or urge phenomena, and 3 = no or minimal control of bladder function) and cognitive deficit (0 = normal, 1 = minimal attention or memory deficits, 2 = considerable attention or memory deficits but oriented to situational context, and 3 = not or only marginally oriented to situational context) was used to characterize and grade the clinical syndrome. Patients were evaluated both before and 4 to 6 hours after the LP.

### Sample collection

All participants underwent LP in the L3-4 or L4-5 interspace to drain 30 ~ 50 ml of CSF to evaluate response to LP from 10:00 to 12:00. During the procedure, 10 ml of CSF was collected in polypropylene tubes after discarding the first 3 ~ 4 ml. Bloody or cloudy samples were rejected. No serious adverse events were reported. The samples were immediately centrifuged for 15 minutes at 2,000 rpm to remove cells and aliquots were stored in polypropylene tubes and immediately frozen at −80°C until analysis. They were thawed just before analysis.

### ELISA methods

CSF T-tau concentration was determined using a sandwich enzyme-linked immunosorbent assay ([ELISA] Innotest hTAU-Ag, Innogenetics, Ghent, Belgium) specifically constructed to measure all tau isoforms irrespective of phosphorylation status, as previously described [22]. P-tau was measured using a sandwich ELISA method (Innotest Phospho-Tau[181P]), as previously described [23]. Aβ42 levels were determined using a sandwich ELISA (Innotest β-amyloid [1-42]), specifically constructed to measure amyloid-β containing both the 1st and 42nd amino acids, as previously described [24]. All biomarker levels were measured in duplicate according to the manufacturer’s instructions. The CSF samples of iNPH patients were analyzed twice using different aliquot.

### Magnetic resonance image

The Evans index and white matter hyperintensity were assessed using MRI. The Evans index was defined as the maximal frontal horn ventricular width divided by the transverse inner diameter of the skull and signifies ventriculomegaly if it is 0.3 or greater [2]. White matter hyperintensity was evaluated by the method designed by Clinical Research for Dementia of South Korea Study (CREDOS). Both periventricular (1-3) and deep (1-3) white matter hyperintensities were assessed [25]. Each longest-diameter white matter change around the lateral ventricles (capping or banding on the periventricular areas) or deep in white matter (especially the centrum semiovale) were evaluated separately. The deep white matter changes were rated as 1 (< 10 mm), 2 (≥ 10 mm, < 25 mm), or 3 (≥ 25 mm) and periventricular white matter changes were rated as 1 (< 5 mm) or 2 (≥ 5 mm, < 10 mm), or 3 (≥ 10 mm). Hippocampal atrophy was graded by Scheltens’ method [26].

### Neuropsychological tests

Neuropsychologists assessed participants’ cognitive functioning via the extensive Seoul Neuropsychological Screening Battery (SNSB) [19] covering five specific cognitive domains, as follows.

(1) The attention and working memory assessment used the digit span forward and backward tests.

(2) The language function assessment employed the Korean version of the Boston Naming Test (K-BNT) [27].

(3) The visuospatial function assessment was the patient’s copy score of the Rey Complex Figure Test (RCFT), neuropsychological assessment in which examinees are asked to reproduce a complicated line drawing, first by copying and then from memory.

(4) Memory function was divided into verbal and visual memory. We assessed verbal memory by means of the Seoul Verbal Learning Test (SVLT), the Korean version of the revised Hopkins Verbal Learning Test (HVLT-R), testing participants on the immediate recall, delayed recall, and recognition tasks. To assess visual memory, we tested participants on the RCFT’s immediate recall, delayed recall, and recognition tasks.

(5) To assess frontal lobe functioning, we used the contrasting program, go-no go, the semantic and phonemic aspects of the Controlled Oral Word Association Test (COWAT) and Stroop test.

### Statistical analysis

We used chi-square, analysis of variance (ANOVA), and Kruskal-Wallis tests to compare demographic data of each group and analysis of covariance (ANCOVA) to compare clinical data and the neuropsychological test percentile scores adjusted by the age, gender and years of education of normative data from general population. Post-hoc analyses with Least Significant Difference (LSD) method were performed for between-group comparisons. Intraclass correlation coefficient was calculated to show the test-retest reliability of ELISA. Receiver operating characteristics (ROC) curve analysis was used to identify the cut-off levels with the optimal combination of specificity and sensitivity. Mann-Whitney test was used to compare the scores of neuropsychological tests between the subgroups of iNPH patients, adjusted for age, sex and duration of education. All statistical analyses were performed using SPSS 13.0 (SPSS Inc, Chicago, IL, USA). Null hypotheses of no difference were rejected if p-values were less than .05.

## Results

### Demographic and clinical characteristics of the subject groups

**Figure 1:**
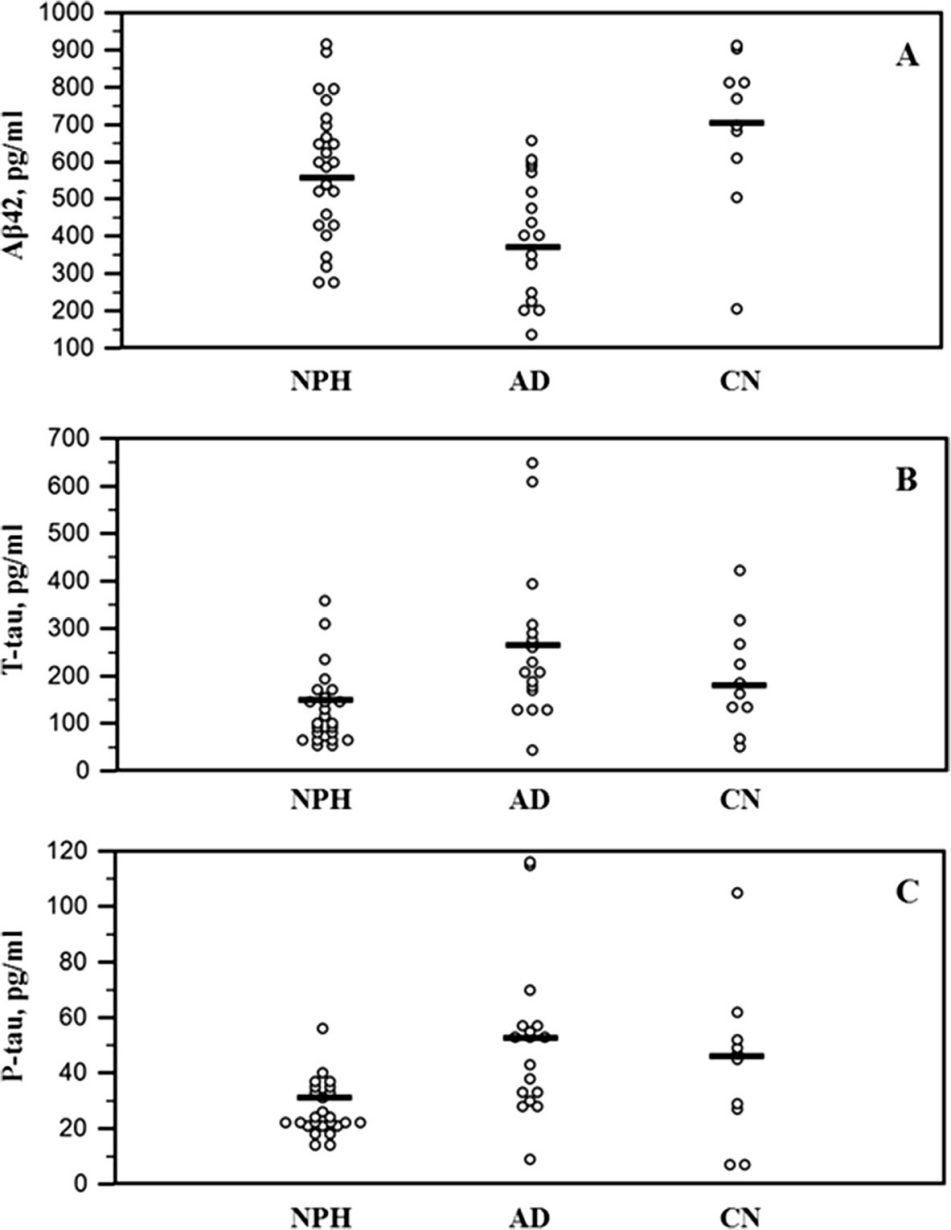
Levels of CSF Aβ42 (A), T-tau (B) and P-tau (C) in the subject groups.

Demographic and clinical characteristics and results of statistical analyses are summarized in Table 1 and plotted in Figure 1. The age of AD group was significantly older than the control group (p = 0.029). The intraclass correlation coefficient of Aβ42, T-tau and P-tau were 0.922, 0.908 and 0.960, respectively. The mean value of the two test results of each analysis was used for statistical analyses. CSF Aβ42 level of AD was significantly lower than iNPH (p = 0.013) and control group (p = 0.001) after adjustment for age, sex and duration of education. CSF T-tau and P-tau levels were significantly higher in AD than iNPH (p = 0.003 and p = 0.002, respectively).

**Table 1:** Demographic and clinical characteristics of the subject groups (N = 52)

	**iNPH**	**AD**	**Control**	**p**_**1**_	**p**_**2**_	**p**_**3**_
**n (M/F)**	25 (12/13)	17 (10/7)	10 (3/7)			
**Age, yr**	73.3 ± 7.0	72.2 ± 10.0	63.0 ± 6.7	0.924	0.029*	0.090
**Education, yr**	8.5 ± 5.2	6.1 ± 5.6	5.5 ± 5.2	0.469	0.498	0.970
**K-MMSE**	19.5 ± 6.9	18.3 ± 2.1	26.6 ± 2.5	0.832	0.042*	0.029*
**Aβ42, pg/ml**	579.8 ± 182.3	409.2 ± 166.1	691.8 ± 212.7	0.013*	0.241	0.001*
**T-tau, pg/ml**	131.9 ± 77.6	259.6 ± 161.5	196.9 ± 114.4	0.003*	0.312	0.382
**P-tau, pg/ml**	27.0 ± 9.6	51.3 ± 28.3	43.0 ± 28.5	0.002*	0.123	0.597

Continuous variables are presented as mean ± standard deviation. iNPH: Idiopathic Normal Pressure Hydrocephalus; AD: Alzheimer’s Disease; M: Male; F: Female; K-MMSE: Korean version of Mini Mental Status Exam; p_1_: p-value between iNPH and AD; p_2_: p-value between iNPH and control; p_3_: p-value between AD and control.

*p < 0.05.

### Classification of the iNPH patients according to ELISA results

**Figure 2:**
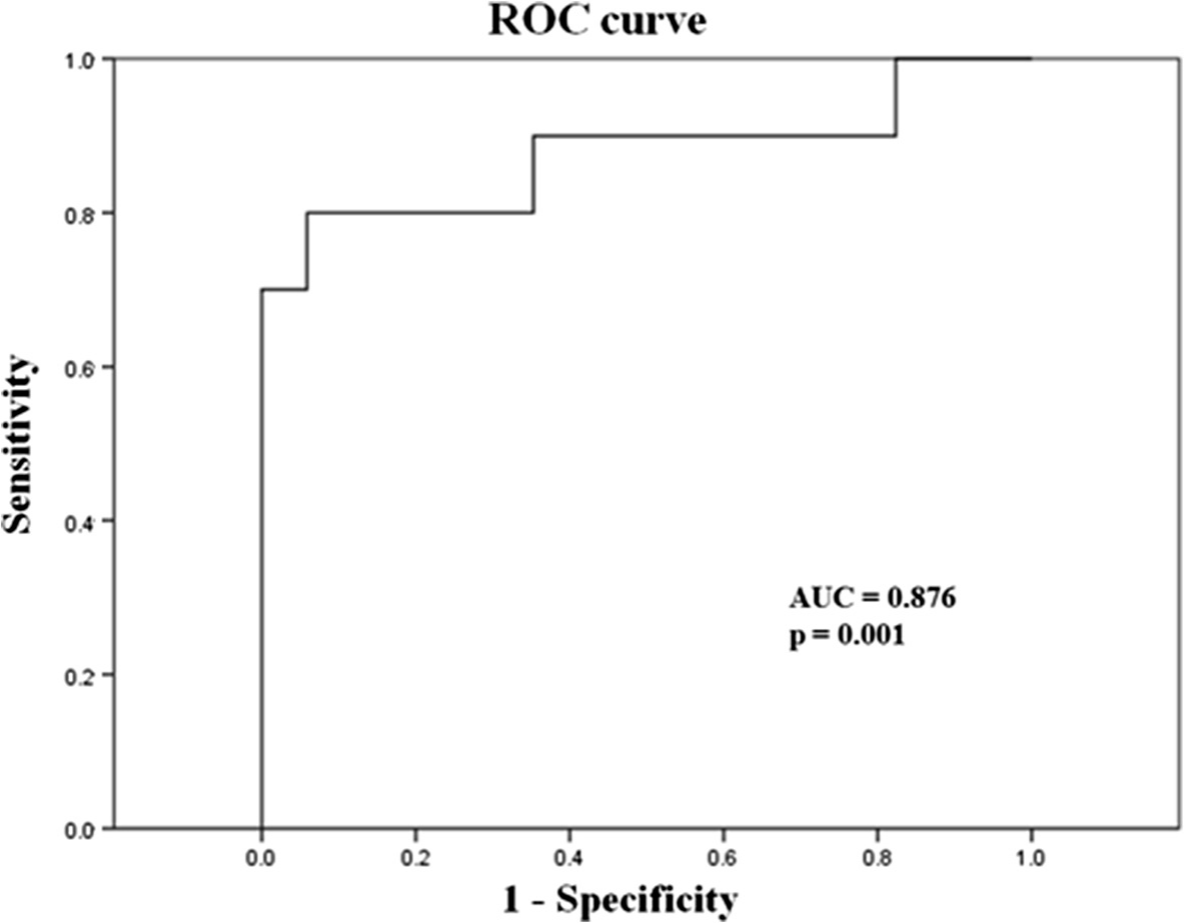
Receiver operating characteristic (ROC) curve of CSF Aβ42 for discrimination between the control and AD patient groups.

To determine a cut-off level of the coexistence of AD in iNPH patients, ROC curve analysis was performed. CSF Aβ42 was used because it was the only biomarker which was significantly different between AD and control group. The area under the curve was 0.876 with p-value of 0.001 (Figure 2). Following recommendations of previous studies, sensitivity more than 85% was selected in the determination of cutoff level of 490 pg/ml, similar to the previous study (482 pg/ml) [28].

### Demographic and clinical characteristics of the iNPH patient subgroups

Eight iNPH patients were classified into the lower CSF Aβ42 group and 17 patients were classified into the higher CSF Aβ42 group. We statistically compared the lower CSF Aβ42 group and the higher CSF Aβ42 group using the Mann-Whitney test. There was no difference in age, sex and duration of education. Among the variables tested, hippocampal atrophy grade was significantly higher in the lower CSF Aβ42 group than the higher CSF Aβ42 group (p = 0.02). There was no difference in the iNPH grading score and improvement rate after LP between the two groups (Table 2).

**Table 2:** Demographic and clinical data of the iNPH patient subgroups by CSF Aβ42 level (N = 25)

	**Lower CSF Aβ42**	**Higher CSF Aβ42**	**p-value**
**n (M/F)**	8 (3/5)	17 (9/8)	0.673
**Age, yr**	76.1 ± 7.3	72.0 ± 6.7	0.187
**Education, yr**	7.7 ± 5.6	8.7 ± 5.2	0.687
**K-MMSE**	16.8 ± 6.6	20.8 ± 6.9	0.193
**Disease duration, day**	724.1 ± 513.6	769.4 ± 520.4	0.857
**Aβ42, pg/ml**	368.7 ± 72.3	679.1 ± 121.7	<0.001*
**T-tau, pg/ml**	145.7 ± 103.9	125.3 ± 64.6	0.551
**P-tau, pg/ml**	28.2 ± 13.8	26.4 ± 7.3	0.682
**Evans ratio**	0.34 ± 0.02	0.36 ± 0.03	0.277
**DWMH**	1.6 ± 0.9	1.5 ± 0.8	0.804
**PVWMH**	2.0 ± 0.7	1.9 ± 0.7	0.856
**Hippocampal atrophy**	1.6 ± 0.9	0.7 ± 0.6	0.020*
**Pre-gait score**	2.75 ± 1.48	2.59 ± 1.54	0.807
**Pre-urinary score**	1.00 ± 1.06	0.94 ± 0.96	0.892
**Pre-cognition score**	1.50 ± 0.75	1.41 ± 0.79	0.795
**Pre-iNPH score sum**	4.13 ± 2.94	3.94 ± 2.86	0.883
**Post-gait score**	2.00 ± 1.69	2.00 ± 1.11	1.000
**Post-urinary score**	0.63 ± 1.06	0.47 ± 0.80	0.689
**Post-cognition score**	1.38 ± 0.91	1.12 ± 0.78	0.474
**Post-iNPH score sum**	3.00 ± 3.38	2.65 ± 2.14	0.753
**Gait score improvement**	0.75 ± 0.70	0.59 ± 0.87	0.651
**Urinary score improvement**	0.38 ± 0.74	0.47 ± 0.71	0.761
**Cognition score improvement**	0.13 ± 0.35	0.29 ± 0.47	0.377
**iNPH score improvement**	1.25 ± 1.48	1.29 ± 0.68	0.950

Continuous variables are presented as mean ± standard deviation. iNPH: Idiopathic Normal Pressure Hydrocephalus; AD: Alzheimer’s Disease; M: Male; F: Female; K-MMSE: Korean version of Mini Mental Status Exam; PVWMH: Periventricular White Matter Hyperintensity; DWMH: Deep White Matter Hyperintensity. *p < 0.05.

### Neuropsychological test results of the iNPH patient subgroups according to CSF Aβ42 level

A total of 18 iNPH patients underwent detailed neuropsychological tests as an initial evaluation before CSF drainage. Seven patients belonged to the lower CSF Aβ42 group and 11 belonged to the higher CSF Aβ42 group. Among the neuropsychological test scores, the lower CSF Aβ42 group had a lower score in Digit span forward test (p = 0.018), RCFT copy test (p = 0.043) and SVLT immediate recall test (p = 0.009) after adjustment for age, sex and duration of education (Table 3, A).

**Table 3:** Neuropsychological test results of the iNPH patient subgroups by CSF Aβ42 level before CSF drainage (N = 18, A) and change before and after CSF drainage (N = 10, B)

	**(A)**			**(B)**		
	**Lower CSF Aβ42**	**Higher CSF Aβ42**	**P**	**Lower CSF Aβ42**	**Higher CSF Aβ42**	**p**
	**(n = 7)**	**(n = 11)**		**(n = 5)**	**(n = 5)**	
**Attention**						
Digit span forward test	4.7 ± 1.1	6.2 ± 1.4	0.018*	0.2 ± 1.3	-0.6 ± 0.8	0.131
Digit span backward test	2.0 ± 1.5	2.7 ± 1.1	0.271	0.0 ± 0.7	-0.2 ± 0.8	0.911
**Language function**						
K-BNT	25.3 ± 6.6	36.1 ± 14.3	0.157	2.4 ± 6.5	0.6 ± 3.6	0.602
**Visuospatial function**						
RCFT Copy	12.8 ± 10.7	23.7 ± 12.5	0.043*	0.1 ± 4.5	-1.0 ± 2.1	0.577
**Memory function**						
SVLT immediate recall	6.8 ± 1.9	13.3 ± 3.6	0.009*	2.0 ± 3.0	4.2 ± 3.8	0.117
SVLT delayed recall	0.1 ± 0.3	1.8 ± 2.0	0.204	0.0 ± 0.0	2.0 ± 1.8	0.054
SVLT recognition	15.5 ± 2.9	15.9 ± 4.0	0.820	-1.0 ± 4.1	1.4 ± 2.8	0.602
RCFT immediate recall	2.9 ± 4.3	7.9 ± 6.7	0.129	-0.8 ± 2.5	2.2 ± 3.5	0.245
RCFT delayed recall	1.8 ± 2.8	6.6 ± 6.2	0.232	0.3 ± 2.3	3.0 ± 3.2	0.245
RCFT recognition	16.0 ± 3.3	16.9 ± 3.1	0.480	-2.6 ± 3.2	2.0 ± 3.0	0.465
**Frontal function**						
Contrasting program	15.0 ± 8.3	14.2 ± 9.1	0.691	-0.2 ± 0.4	0.2 ± 0.4	0.180
Go-no go	9.6 ± 10.2	11.0 ± 9.1	0.499	3.2 ± 8.9	2.8 ± 6.2	0.451
Semantic COWAT	6.3 ± 3.9	8.8 ± 6.1	0.269	-1.4 ± 3.3	5.4 ± 6.1	0.117
Phonemic COWAT	10.0 ± 6.0	14.4 ± 11.4	0.926	-1.0 ± 1.7	5.4 ± 6.1	0.018*
Stroop test: color reading	26.0 ± 13.2	44.5 ± 35.9	0.905	-4.4 ± 9.5	8.2 ± 8.1	0.008*

Continuous variables are presented as mean ± standard deviation. iNPH: Idiopathic Normal Pressure Hydrocephalus; AD: Alzheimer’s Disease; K-BNT: Korean version of Boston Naming Test; RCFT: Rey Complex Figure Test; SVLT: Seoul Verbal Learning Test; COWAT: Controlled Oral Word Association Test.

In a subset analysis of 10 patients who underwent follow-up neuropsychological tests after CSF drainage, the lower CSF Aβ42 group showed significantly less improvement in phonemic COWAT (p = 0.008) and color reading test in Stroop test (p = 0.018) after LP compared to the higher CSF Aβ42 group after adjustment for age, sex and duration of education. The mean interval between the first and the second neuropsychological test was 13.2 ± 9.4 days (Table 3, B).

## Discussion

Although there have been a few studies about the coexistence of AD in iNPH patients and its clinical implication, to the best of our knowledge, this is the first study which reports the difference in detailed neuropsychological tests before and after CSF drainage according to CSF biomarkers for AD. The major findings of this study were as follows: (1) All three biomarkers for AD diagnosis were significantly different between AD and iNPH. (2) There was no difference in demographic and clinical characteristics including the response to CSF drainage using iNPH grading system between the lower CSF Aβ42 group and the higher CSF Aβ42 group. (3) Comparison of initial neuropsychological tests showed deficits in attention, visuospatial function and verbal memory are more prominent in the lower CSF Aβ42 group than the higher CSF Aβ42 group. (4) Neuropsychological performance improvement before and after CSF drainage were significantly less in phonemic categorical naming and frontal inhibitory function in the lower CSF Aβ42 group than the higher CSF Aβ42 group.

In the comparison of Aβ42, T-tau and P-tau between iNPH and AD, Aβ42 was lower and T-tau and P-tau were higher in AD than in iNPH. These results correspond well with earlier studies which reported various CSF biomarkers indicating less AD pathology in iNPH patients than AD patients [11,29,30]. After determination of cutoff level of AD using ROC analysis of CSF biomarkers in AD and control group, 8 out of 25 iNPH patients (32%) were classified as having concomitant AD. This is consistent with the overall prevalence of AD in normal elderly population [31].In contrast to the consistent low Aβ42 level in iNPH, there have been inconsistent reports regarding the T-tau and P-tau levels. Some recent studies using ventricular CSF during shunt procedure or external lumbar drainage (ELD) reported higher T-tau and P-tau level in iNPH patients [10,14,15].On the other hand, a recent report investigating various biomarkers in lumbar and ventricular CSF showed lower T-tau and P-tau level in iNPH than normal control group [16]. Elevated T-tau level has been detected in various diseases causing neuronal injury such as stroke, trauma, hemorrhage and encephalitis [32-34]. Shunt operation and ELD procedure might result in neuronal injury and elevated T-tau and P-tau level in these studies. Cortical neuronal injury from the advanced stage of iNPH and concomitant AD might affect this discrepancy, as well.

Except for the hippocampal atrophy, all the radiological and clinical symptomatic indicators of iNPH patients including response to CSF drainage did not differ between the two subgroups divided by CSF Aβ42 level. These results are similar to those of previous studies which reported that iNPH patients benefit equally from shunting regardless of the presence of AD pathology [7-9]. Recently a study performed to compare CSF biomarkers and response to ELD showed that none of the biomarker predicted the ELD results [14]. However there have been a few reports that cortical AD pathology and ventricular CSF biomarker for AD resulted in a less robust response to shunting [4,10]. Further long-term follow-up study using simultaneous investigation of AD biomarkers of cortical pathology and ventricular and lumbar CSF might provide some of these answers.

Although there was no difference in general cognitive function between the two subgroups, the initial detailed neuropsychological tests revealed more deficits in attention, visuospatial function and verbal memory in the lower CSF Aβ42 group. These domains are well known to be impaired in the early course of AD. Therefore these results suggested that AD pathology may impact an additive AD-related cognitive dysfunction in iNPH patients regardless of underlying cognitive dysfunction caused by iNPH. In addition to the initial difference, categorical naming and inhibitory executive dysfunctions in the lower CSF Aβ42 group, which were considered as iNPH-related cognitive dysfunction, were less improved after lumbar puncture compared to the higher CSF Aβ42 group. These results suggested that some portion of the frontal lobe dysfunction in the lower CSF Aβ42 group was possibly caused by the concomitant AD and not improved after lumbar puncture. In contrast, the frontal lobe dysfunction in the higher CSF Aβ42 group was solely caused by the pathophysiology of iNPH and improved more than the lower CSF Aβ42 group. This pattern of improvement after lumbar puncture was not reported before but the similar pattern of post-shunt improvement was noted in a previous study which observed improvement of subcortical dysfunction after shunting [10].

We assumed that memory impairment and visuospatial dysfunction in iNPH were mainly caused by concomitant AD which was defined by lower CSF Aβ42. This assumption was usually correct in pure AD but the correlation of the cognitive dysfunction with CSF biomarkers is more complicated in iNPH. For example, in a previous series of 17 iNPH cases, the visuospatial dysfunction was the prominent cognitive feature which was against the classic view of iNPH as a subcortical, frontal type of dementia [35]. Furthermore in a recent study, the decreased CSF Aβ42 was not considered as a biomarker for concomitant AD but a consequence of a reduced brain metabolism secondary to the changed CSF dynamic in iNPH [16]. However, there was neuropathological evidence that the CSF biomarkers for AD correlated with cortical brain biopsy findings indicating AD [17]. There were also studies showing that the memory deficit as the leading symptom and cortical biopsy findings predicted later development of AD in iNPH [36,37]. Further studies are warranted regarding the correlation between the cognitive symptoms and CSF biomarkers for AD in iNPH.

We should accept that this study has several limitations. One limitation of this study is its relatively small sample size. Another limitation is lack of analysis of long-term follow-up data such as shunting history. The authors also acknowledged that the possible iNPH criteria is inclusive and the elevated T-tau and P-tau levels might be induced by some different circumstances. In addition, normal control subjects were recruited from the clinic and possibly had cognitive complaint more than those recruited from the cohort. Subjects with cognitive complaint are known to have more AD pathology than those without it [38]. This may affect the lack of difference in T-tau and P-tau between AD and control groups. The improvement after lumbar puncture may be affected by the learning effect but there was a report that no learning effect was found in patients with iNPH on any of neuropsychological tests [39].

## Conclusions

Our study suggested that concomitant AD in iNPH patients might contribute to lumbar puncture or shunt unresponsiveness, especially in the field of cognitive dysfunction.

## Competing interests

There are no financial or non-financial competing interests on this study.

## Authors’ contributions

TSL, JYC, SAP, YCY, HYL, and SYM carried out the study, analyzed the data and produced the draft. BGK, ISJ and KH improved on the draft and also helped with the recruitment of patients. All authors read and approved the final manuscript.

## Pre-publication history

The pre-publication history for this paper can be accessed here:

http://www.biomedcentral.com/1471-2377/14/66/prepub
